# Extracts and Gleanings

**Published:** 1875-09

**Authors:** 


### Extracts and Gleanings.

*Incontinence of Urine.*—Dr. F. N. Otis, of New York, writes to *The Lancet:* One of your correspondents asks for infomation as to the treatment of incontinence of urine, and half a dozen responces are made. A little more careful perusal of the department of *The Lancet* devoted to "Notes, Short Comments, and Answers to Correspondents," would have caused some consideration of the note on "Ritual Circumcision," in the issue of Jan. 9th, where Dr. A. Mayer says: "It is also a known fact that circumcision cures enuresis nocturna, when caused by a prepuce with a narrow opening." The fact that of the six respondents, two recommend belladonna, and the other four as many different modes of managing the difficulty, would show that the *cause* had not been looked after. My own experience, which has been extensive, has convinced me that enuresis in youth and in adult life is frequently due to a contraction either of the preputial orifice or of the meatus urinarius, and that sedatives and narcotics may palliate, but do not cure. Circumcision, or division of the preputial orifice, when the prepuce alone is at fault, will give prompt and permanent relief; when this does *not* give relief, the meatus urinarius will often be found constricted, and a relief of this constriction will give the desired result. Let me cite a case or two:

G. H———, a lad of ten, had been troubled with nocturnal incontinence two or three times in the night, and also often during the day, for two years. Belladonna, in quarter-grain doses, relieved him temporarily, but on cessation of the drug would return. He was found to have redundant prepuce with contracted orifice. I circumcised him in October, 1874, found some adherence of prepuce to glans, and also a very small meatus urinarius. This I enlarged to 22 Fr. From date of operation he was completely relieved for four weeks, when the difficulty reappeared. I then made another examination, and found the meatus recontracted to 16 Fr. I then redivided it to 24 Fr. Since then he has had not the slightest trouble, and, furthermore, has been in better health than for several years.

I have just received a letter from Dr. C. H. Mastin, of Mobile, Alabama, which I desire to add to this statement, and let your corespondents judge of its value. Dr. M. is one of the leading surgeons of the Southwest: "Following I furnish you the brief notes of a case which I fancy will interest you, as it bears so strongly on your theory of reflex irritation, which I, for one, believe is ere long destined to become a fixed surgical truth. On the 29th of last month (November, 1874), I was requested to see a little boy, eighteen months old, the son of a gentleman of this city. The child was very delicate, pale, fretful, no appetite, languid, complained of pain whenever he passed his water. He was wakeful at night, and in the nightly habit of wetting his bed during sleep. From the description given me I was led to believe the child had stone. Upon examination, however, I failed to discover stone, but found that the child had an elongated prepuce, the orifice of which was very small, congenitally contracted, so small, indeed, that there was difficulty in passing an ordinary silver probe. I was satisfied that the case was one of reflex irritation from narrowing of the preputial orifice. The physician with whom I saw the case, in consultation, laughed at this diagnosis; but, inasmuch as there was an evident obstruction to urination, consented to a free division of the preputial orifice. The results of this simple operation proved the correctness of your views and my diagnosis established thereon. The very next day my little patient was decidedly improved, ate well, slept quietly, had no incontinence of urine, and up to to-day (one month from the date of operation) he has remained perfectly well in this respect, and his father says he has gained eight to ten pounds of flesh, and is in better health than ever before."—*Medical and Surgical Reporter.*

*Puerperal Fever.*—There is no subject more important than the causes of puerperal fever, and perhaps none about which such a diversity of opinion prevails. The contagionists and non-contagionists have debated the subject for years, and no conclusion has yet been reached. Fortunately for women, all obstetricians are doubtful, and give their patients the benefit of the doubt. The following case is very clear, and seems to give the key to this terrible problem:

Mr. Spencer Wells, in his address on puerperal fever before the Obstetrical Society of London, said: "A country surgeon attends a man who has erysipelas after a broken arm. He also attends a healthy woman in an isolated cottage in a natural labor. There is no puerperal fever in the district, yet this woman dies of puerperal fever. . . . Such a history as this would have tenfold weight, as being free from numerous sources of fallacy and doubt."

Mr. S. N. Squire, of Wivenhoe, reports (*British Med. Journ.*, May 22, 1875), a case which occurred in his practice which presents the conditions prescribed: "On the night of February 11th last, my assistant went to attend a man who had fallen down and cut his head open over the occiput down to the bone. The wound was about an inch long. On the second night it bled, evidently from a small artery, which he arrested by a compress of lint. On the 20th the man was taken very ill. I myself went to see him, and found that he had been suffering from rigors. I examined the wound; the scalp was somewhat swollen. I carefully washed and dressed the part. The same evening I was called to attend a woman in confinement (age about 37, fifth child), who had a natural labor. The next day I found that the man was suffering from erysipelas; it was running down over the forehead. On the 22d the woman had a chill, with all the symptoms of puerperal fever setting in. She died on the 27th; the man likewise died on March 1st. I took every precaution, whilst attending other cases, and did not wear the same external clothing; so I did not affect any other lying-in-woman. Early on March 3d I attended another (age 22, second confinement), who, on the day following, had all the symptoms of the previous case. I questioned the nurse, as she came from the village where the man died, whether she had been in the house. She informed me she had been there to assist, and left that place direct to go to the woman in labor. That case terminated fatally on the 10th. The child had erysipelas at the navel, which spread all over the body; it died on the 18th. A nurse, who was in almost constant attendance upon the man, had an abrasion on the nose; she had erysipelas on March 3d, and died on the 7th. Whilst attending the man, I was also daily dressing two women, each for an ulcerated leg; both had erysipelatous inflammation of the leg. The husband of one, an old man, aged about 78, had erysipelas over the head and face, from which he got better, but died of exhaustion on May 6th. A son of the old man's master called to see him on April 25th; he had a slight scratch on the septum oi the nose. On the 27th erysipelas made its appearance, and spread over the face, from which he has now recovered.

"To go back again to the first case. On March 13th a woman, who had been several times to see the man (whose house was directly opposite her own), had a severe attack of erysipelas over the head and face; she recovered. A young woman likewise visited her, and at the same time had her ears pierced for rings; erysipelas affected them, and spread rapidly over the head and face. She recovered after a severe attack. During the interval of the two puerperal cases I attended other women, who escaped infection. Thus, I had nine cases of erysipelas and two of puerperal fever, with six deaths, all to be traced from the first. In looking over my midwifery list, I find I had previously attended 1139 cases without losing one. I have been in practice twenty-five years, and, during that time, I only lost four cases, not including the last two I have previously mentioned. One died of inflammation of the lungs, three days after confinement; another (a turning case), of peritoneal inflammation, three weeks after confinement; another of scarlet fever; and the fourth of puerperal fever, the cause of which I could not discover. I generally make it my practice, if I have any contagious disease about, to visit my childbed cases first; the doing so, I think, is one reason why my death-rate is so low. Idiopathic erysipelas I consider not contagious; but traumatic, being caused by pyæmia, I think is, and I hold that it would be very unwise to attend a labor directly after visiting such a case. I have had many cases of the former kind, but cannot now remember losing one, nor have I seen that another person has taken it from one so affected."

May not the remark of Mr. S., that he has never seen any ill effects follow idiopathic erysipelas, solve the problem? In a simple case of erysipelas, we have no raw surface to give off septic germs. In traumatic erysipelas, on the contrary, every thing is in a favorable condition for their evolution. We all know the protection that a sound epidermis gives to the nurses or dressers in our hospitals, and if this membrane is so potent in keeping out poisons, must it not be equally so in keeping them in, when generated in the system?

H. B. L.

*A New Instrument for Operating on Vesico-Vaginal Fistula.*—Dr. William A. Byrd, of Quincy, Illinois, gives a description of a new instrument for simplifying this operation. Finding great difficulty from the prolapse of the wall of the bladder in large fistulæ, he devised the following instrument for its prevention: Take two ordinary rubber balloons, such as you find in all toy-shops; insert one balloon into the other, to give the required strength. Insert a piece of hard rubber tubing, three-fourths of an inch long and three-sixteenths in diameter, into a piece of elastic tubing same diameter and seven inches long; over both of these the neck of the balloon is fastened. The instrument is used by passing the long tube through the fistula into the urethra, and in that way placing the bag in the bladder; it is then filled with cold water. To withdraw it after the operation, you have only to allow the water to escape, and gently pull on the long tube. He claims the following advantages from its use:

1. The material is to be had in almost any town, and will not cost above forty or fifty cents, and one with a little ingenuity can make one in from five to ten minutes. 2. It is easily dilated with any ordinary syringe, when it defines the fistula perfectly, throwing the walls of the fistula prominently out from the convex surface of the balloon, which renders the paring more speedy, certain and easy. 3. It prevents the posterior wall of the bladder from interfering with the operation. 4. It prevents blood flowing into the bladder, and there clotting and giving trouble. 5. It allows the sutures to be more rapidly inserted, and more easily placed at proper distances from each other. For a like purpose a good housewife places a ball in a stocking she is going to darn. 6. By pressure and the temperature of the distending fluid, it represses hæmorrhage. 7. When undistended it occupies a very small space. 8. At the suggestion of my friend Dr. Landon, of Burton, Illinois, it can readily be applied as a tampon in cases of metrorrhagia, possessing the advantage of being smaller, cheaper, and more readily obtained, than Baun's colpeurynter.—*N. Y. Medical Journal.*

*On Extraction of Cataract by a Median Section through the Cornea.*—The editor of *La Cronaca Oftalmológica*, of April 12, gives a short account of a method of operation which has been practiced since 1869 by his fellow-countryman, Dr. Vicente Chiralt, for the extraction of cataract, and with very marked success. The operation consists of three stages, the first and most important of them being, the cutting a section transversely across the cornea at the junction of the middle with the lower third, by means of a Von Graefe's knife, which is entered at the selero-corneal margin and is passed rapidly across the anterior chamber, and made to emerge on the opposite and corresponding point. No iridectomy is performed, and chloroform is considered unnecessary. The capsule is opened in the usual way as the second stage of the operation, and the lens is generally removed without difficulty with the aid of slight pressure upon the upper lid, assisted by Von Graefe's elastic spoon.

Dr. Chiralt considers the operation a complete success when the patient can read No. IV. Snellen ; moderately successful when some letter larger than No. IV. is read ; and unsuccessful when the largest types only can be read. When measured in this way, his results may be thus stated :—

| | | | | | |
|---|---|---|---|---|---|
| Good, | when the patient could read S. I. V. | | | . . . | 73 |
| Moderate, | " | " | " | some larger type . | 7 |
| Bad, | " | " | " | only S. C. or C. C . | 5 |
| | | | | | — |
| | | | | | 85 |

The editor admits the similarity of this operation to that proposed and practiced by Le Brun ; but he claims for Dr. Chiralt the priority of its performanee.—*Lond. Medical Record*, June 23, 1875.

*An Improved Method of Treating Certain Cases of Cataract requiring Extraction.*—Mr. J. Vose Solomon, Surgeon to the Birmingham Eye Hospital, states, (*Lancet*, July 31, 1875) that in some ophthalmic works which discuss the cure of senile cataract, the presence of a deep-set eye is given as a reason for the selection of depression or reclination in preference to the operation of extraction. He imagines no surgeon who has had experience in such cases will deny that a deep-set eye occasions some embarrassment in the making of the corneal section with a Graefe's or Beer's knife, especially where anæsthetics are not employed.

In a recent case at the Birmingham Eye Hospital, Mr. Solomon improvised a simple and effective method of overcoming the difficulty under consideration by making, with a pair of sharp scissors, a perpendicular incision through the substance of the lower lid of about three-eighth of an inch in length and one-sixth of an inch from the commissure. Steady continuous pressure by means of a small sponge restrained the bleeding within innocent limits. The front of the eye having been now made easy of access to the knife, the section of the cornea was completed without difficulty, and the lens safely delivered without accident. No anæsthetic was employed. The lid was not sutured, but dressed with cerate instead of dry rag, and two turns of a narrow domett roller. The patient, a collier or sixty-eight years, recovered without a single painful or unfavorable symptom, and was discharged cured on the fourteenth day of the operation, with a clear black pupil, good vision, and perfect cicatrization of the tarsal cartilage.

The above plan should have preference to any that interferes traumatically with the condition of the commissure, from the impossibility of its inducing abnormal eontraction of the palpebral aperture or inversion of the lid (entropion)—accidents of no mean importance.

The operation of *reclination* has fallen into undeserved neglect, and, as a consequence, eyes are destroyed which would have had a good chance of vision if it had been selected for performance instead of extraction. A resort to it would be advisable where there is extensive fatty degeneration of the cornea and other tissues, and in subjects in whom the arteries have become seriously spoilt by atheroma, also in those who are affected with tertiary syphilis of old date. In the last two pathological conditions, intraocular hemorrhage is the complication to be dreaded after the removal of the lens, or even upon the mere evacuation of the aqueous humour (no hypothetical case); in the first death of the corneal flap within eighteen hours of the performance of the extraction is not uncommon.—*Abstract of Monthly Science.*

*A New Method for Healing Ulcers.*—Dr. Nussbaum claims to have successfully treated upwards of sixty cases of chronic, extensive, and otherwise intractable leg ulcers, by the following simple procedure:

The patient is at first etherized, and then around the ulcer of the leg or foot, a finger's breadth from its margin, an incision extending down to the fascia is made; numerous blood-vessels are divided, and a severe hemorrhage ensues, unless a fine pledget of lint be packed into the cut and the entire ulcer strongly compressed. The packing with lint is also necessary to prevent union of the cut edges by the following day. Upon the second day, the bandage and lint are removed; from then until a cure is effected, a simple water dressing is applied.

The author states that an astonishing change can be seen, even in the first twenty-four hours. The ulcer which yesterday threw off quarts of thin, offensive, ichorous pus, furnishes to-day not more than a tablespoonful of thick, non-offensive, healthy pus. The old ulcer becomes rapidly smaller, healing from the margin towards the centre, and is healed up in a short time; but the cut is changed into a broad circular sore, which also rapidly cicatrizes.

The great diminution of the secretion, and other favorable changes occurring in the ulcer, find an explanation in the fact, that the circumcision has divided dozens of large, abnormally widened blood-vessels. Time is thus given for the lessened nutritive material, which was previously carried off by the excessive secretion, to be transformed into cells and connective tissue; in other words, granulations are formed, which fill up and heal the deep ulcer. Without claiming this as a radical method, the author assures us that the cure is much more rapid and the cicatrix becomes more elastic and resisting, than in ordinary means applied, which usually require so much time that the patients depart with half-cured ulcers, soon finding themselves in their previously deplorable condition.—*Philadelphia Medical Times.*

*Diphtheria of a Vaccination Pustule followed by General Diphtheria* (Dr. Ludwig Letzerich: *Virchow's Archiv*, B. lxiii.).—The patient in this case was a child aged four months, which, until vaccination, had been perfectly healthy. On the thirteenth day after this operation was performed, an erysipelatous swelling developed itself about the wound, and spread with great rapidity over the entire body. Œdema soon appeared, followed by icterus and petechial exudations, and death took place on the twelfth day of the disease, the twenty-fifth from the day on which vaccination was performed. At the post-mortem examination lesions characteristic of diphtheria were found, and the question arose whether the lymph which had been used had been the conveyer of the disease, or whether the diphtheria had been contracted at the wound, but at a later period. The fact that diphtheria in such cases makes its appearance some days after vaccination has been performed would seem to indicate that it is not inoculated with the lymph; but, to make this point more clear, experimental inoculations on animals were made. From these the following conclusions were drawn:

1. Vaccine which is infected with the organisms which are characteristic of diphtheria, is not capable of causing vaccine pustules.

2. The inoculation of lymph which contains such organisms is rapidly followed by local deposits and general diphtheria.

And these experiments show especially:

1. That the lymph which was used in this case was not thus contaminated, or there would not have been a development of a vaccine pustule at the proper time.

2. That the local diphtheria changes, followed by the disease itself, must be regarded as processes of secondary character.—W. A. *in Medical Times.*

*Diabetes.*—A complete cure of this affection is related in the *Bahia Medical Gazette,* by means of phenic acid, two and a half grains night and morning, in a vehicle of mint-water. The patient, a teacher of music, had been struck from his saddle by a falling tree. Trommer's test had shown a very large amount of glucose or grape sugar in the urine.—*The Doctor, Aug.* 1*st.*

*Heart Diseases—Treatment.*—Dr. J. Milner Fothergill (*London Lancet*) concludes an article on the treatment of diseases of the heart by the following:

(1) It is of the utmost importance, during primary disease of the heart, to reduce its action to a minimum. Hence only easy labor must be attempted. Rest in bed is often desirable.

(2) When dropsy is present, much relief can often be afforded by unloading the distended venous system.

(3) In all cases, the heart should be acted upon directly by agents which increase the vigor of the ventricular contractions, of which digitalis is chief. In properly selected cases this agent may be given uninterruptedly for years without any so-called cumulative action.

(4) The nutrition of the heart should be maintainad by good food and iron, etc. Improvement in the general condition facilitates the action of the special remedies.—*Detroit Medical Review and Pharmacy.*

*Hygiene of Incurable Heart Disease.*—Dr. C. M. Durrant (*British Medical Journal*) gives the following judicious advice on this topic:

(1) All sudden or hurried motion must be avoided.

(2) Prolonged traveling by railroad is highly prejudicial.

(3) Meals should be small in quantity, of easy assimilation and frequent repetition.

(4) Cold liquids should be sparingly taken.

(5) Tea and coffee, in moderation and not too strong, are not injurious.

(6) Sexual intercourse should be absolutely forbidden.

In a word, everything which exhausts nerve force, or draws largely upon it, should be avoided.—*Detroit Medical Review and Pharmacy.*

*Some of the Therapeutic Properties of Jaborandi.*—Mr. Gubler, in presenting the Therapeutic Society of Paris with a sample of Jaborandi, pilocarpus pinnatus, also gives an account of the medical applications of the plant.

He says in anasarca, with effusion in the serous-cavities, the infusion of the leaves gives satisfactory results, the œdema diminishing and the effusion being re-absorbed.

In severe influenza with violent headache, the sialogogue and sudorific effects of the Jaborandi greatly relieves the symptoms of this affection, and shortens its duration.

In asthma he has succeeded in five cases in aborting the attack by giving an infusion of the leaves; relief being obtained as soon as its sialogogue and sudorific effects appeared.

In sub-acute rheumatism he has found the Jaborandi to diminish the pain.

He has also found it of service in ophthalmia because of its sialogogue properties and in polyuria on account of its sudorific properties — *Journal de Therapeutique, M. Gubler,* 10*th April,* 1875.—*New Orleans Medical and Surgical Journal.*

*A Case of Lymphorrhagia.*—The following case is reported by Prof. Petters. For four years a woman of twenty-three had noticed a white, wart-like prominence on the left labium majus, which gave issue to a milky fluid, the flow persisting in spite of various remedies. This was soon complicated by swelling of the left labium and thigh. The patient married a year afterwards. During the ensuing pregnancy the discharge ceased, to recur again after convalescence from an abortion, which occurred at three months, and was followed by peritonitis. During its cessation the inguinal region and left thigh became more swollen. On admission the left thigh measured more than the right; the glands of the left groin were enlarged, and the labium of the same side presented a swelling as large as a pigeon's egg, studded with warty knots, which secreted a milky alkaline fluid, containing albuminate of soda, red blood corpuscles, numerous lymph corpuscles and free fat, but no sugar and no urea. The quantity amounted sometimes to nearly six ounces in an hour and a half. The diagnosis of lymphorrhagia was confirmed by the post-mortem, the patient's death occurring rather suddenly with febrile symptoms and lymphangitis. There was found diffuse peritonitis, with purulent foci in the ovaries and tubes. The inguinal glands were much enlarged, and contained cavities filled with lymph. Around them were also found extensive spaces with smooth walls, likewise filled with lymph. The lymphatics of the left thigh were dilated, and the spleen enlarged.—*Prag Vjhrschr.* cxxv. p. 69, 1875.—*Schmidt's Jahrb.*, 160, ii. June 17, 1875.

*Indian Hemp in Flooding.*—At a meeting of the Edinburgh Obstetrical Society, Dr. Ritchie remarked:

"In cases where flooding set in after delivery, a full dose of the tincture of Indian hemp (*m*xx.) has, in every instance, acted rapidly, checking the loss in a few minutes, even when ergot has failed. I have also found that it possesses the power of controlling and relieving metrorrhagia and profuse menstruation in a marked degree. What the rationale of its action is I do not know."

This is not altogether new in this country.

*Ointment for "Snuffles" in Infants.*—The following is recommended:

| | | | |
|---|---|---|---|
| ℞.—Tannin, | . . . . | gr. $\frac{3}{4}$. | |
| Axungiæ, | . . . . | ʒj–℈j. | |
| Tinct. vanillæ, | . . . | gtt.v. | M. |

After having prepared this ointment, it is applied by rolling between the thumb and index finger a very small square of paper, so as to form a not very rigid cylinder, which will yield easily to any lateral movement which may be made by the infant while it is being introduced into the nostrils. Then, after having smeared the exterior with the ointment, it is introduced deeply into each nasal fossa.—*Medical and Surgical Reporter.*

*The Influence of the Use of Wind Instruments on Chest Affections.* Dr. Burg, a French physician, has published a little book in which he endeavors to controvert, by reference to his own observations and personal experience, the notion commonly entertained that the use of wind instruments is injurious to individuals characterized by pectoral weakness. He remarks: "Many philanthropists, on seeing our young military musicians wield enormous wind instruments, have sorrowed over the few years the poor fellows have to live. Well, they are mistaken. All the men whose business it is to try the wind instruments made at various factories before sending them off for sale, are, without exception, free from pulmonary affections. I have known many who, on entering on this calling, were very delicate, and who, nevertheless, though their duty obliged them to blow for hours together, enjoyed perfect health after a certain time. I am myself an instance of this. My mother died of consumption; eight children fell victims to the same disease, and only three of us survive—and we all three play wind instruments. The day is not far distant, perhaps, when physicians will have recourse to our dreaded art in order to conquer pulmonary diseases.—*Med. Record.*

*Diet of Infants affected with Acute Intestinal Catarrh* (*The Boston Medical and Surgical Journal, August* 12, 1875).—Dr. R. Demme, after an extended experience, recommends as the most appropriate food for infants affected with gastric or intestinal catarrh, the following diet: Add from a quarter of a pound to a pound of beef, freed of fat, to two quarts of cold water, and let this stand from half an hour to an hour; then boil down to one pint; after cooling, skim off fat from the top, and filter. At each time of using (every two or three hours) without being warmed, this is to be mixed with freshly-prepared rice or barley-water. In the intervals of meals the latter should be given alone, best without sugar, to relieve the thirst. If this food is refused, he gives a drink made with the white of an egg, using according to age from one to three eggs in half a pint to a pint of water which has been previously boiled and cooled down to 98°. If the child's strength begins to fail, brandy in doses of from five to thirty drops is added to the rice or barley-water, from three to five times a day. With older children a mixture of milk with the rice-water or barley-water may be tried.

*Glycerin in the Treatment of Diabetes.*—M. Garnier has addressed a note to the Académie des Sciences on the employment of glycerin in the treatment of glycosuria. M. Schutzen, of Dorpat, has shown by his researches that glycerin either alone or associated with tartaric acid, and taken in the dose of half an ounce to an ounce and a half in the course of twenty-four hours, constitutes a valuable adjuvant to the régime especially adapted to glycosuria. M. G. has himself made use of purified glycerin, and has made it more palatabe by mixing it with a certain amount of alcohol and of aromatic substances (mint, anise, bitter orange.) The employment of glycerin has aided him greatly as well as other patients. *Bull. Gén. de Thérap.*, No. 10, 1875.

*Poisoning by Strawberries.*—Dr. Garnier relates a case of fatal poisoning by strawberries (in the *Lyon Medical for June 20th*), occurring in a strong, healthy girl, fifteen years of age. Three hours after eating a cup of berries, "he found her in a dying state, comatose, with complete prostration, livid countenance, and a very small, intermitting pulse." All remedial measures signally failed. The writer has seen several similar cases, and has saved four by emetics, but he has lost more than he has saved. Enormous meteorism is speedily produced, and in consequence thereof, in Dr. G.'s opinion, after two hours, emetics are of no avail; instead of emetics, then, ammonia should be given.—*Chicago Medical Journal.*

*The Dyspepsia of Smokers.*—Dr. Peters, of Paris, alleges that smoking gives rise to a peculiar form of flatulent dyspepsia, characterized by the fact that in such patients charcoal-powder produces no effect. Above all, it is necessary to diminish and forbid the use of tobacco. Pretty good results are obtained from the use of strychnine, especially from the tincture of nux vomica. But these are difficult of administration, and, besides, patients know their names, and fear them. He therefore recommends the following prescription:

℞.—Bitter drops of baumé, . . . . . . ʒj.

Tincture of rheubarb, . . . . . . ʒv.

Make into forty pills, one or two of which are taken before each meal.—*Surgeon and Medical Reporter.*

*The Local Treatment of Lichen Urticatus.*—This skin-disease, specially affecting children, is characterized by wheals, papules, and severe itching, worse at night, but independent of any discoverable parasite. It is treated by Dr. Mackey with an ointment consisting of equal parts of calomel ointment and extract of belladonna, or with one made according to the following formula:

℞.—Storacis, . . . . . ℥j.
Ceræ flavæ, . . . . gr. cxx.
Olei olivæ, . . . . . fl. ℥ss.
Misce secundum artem.

*Syphilitic Ghosts.*—A correspondent of the *British Medical Journal*, writing from Vienna, reports Prof. Zeissl as saying at his clinic: "Some think, when a patient has for some time enjoyed immunity from manifestations of syphilis, that he is cured; but I tell you, gentlemen, that if a man contract syphilis he will die syphilitic, and at the day of judgment his ghost will have syphilis!"

